# Association of a structured prevention algorithm with nurse knowledge and device-related pressure injury severity in critical care: A pre‑post study

**DOI:** 10.1371/journal.pone.0359842

**Published:** 2026-10-27

**Authors:** Yarkaram Zamani Shiva, Fateme Hasandoost, Malihe Nasiri, Tayebeh Mahvar

**Affiliations:** 1 Student Research Committee, School of Nursing and Midwifery, Shahid Beheshti University of Medical Sciences, Tehran, Iran; 2 Department of Medical Surgical Nursing, School of Nursing and Midwifery, Shahid Beheshti University of Medical Sciences, Tehran, Iran; 3 Department of Pediatric Nursing, School of Nursing and Midwifery, Shahid Beheshti University of Medical Sciences, Tehran, Iran; 4 Faculty of Nursing and Midwifery, Kermanshah University of Medical Sciences, Kermanshah, Iran; University of Health Sciences, Beyhekim Training and Research Hospital, TÜRKIYE

## Abstract

**Background:**

Medical Device-Related Pressure Injuries (MDRPI) are a serious complication in intensive care units (ICUs), yet structured prevention algorithms are lacking.

**Methods:**

This single-group, pre-test/post-test quasi-experimental study was conducted in the General Intensive Care Unit from February 2025 to April 2025. The sample comprised 38 nurses and 77 patients (39 pre-intervention, 38 post-intervention). Nurses’ knowledge was assessed using the MDRPUKQ questionnaire, and patient outcomes were monitored using baseline and post-intervention data on pressure injury prevalence and severity. Data were analyzed using SPSS version 26, employing descriptive and inferential statistics including paired t-tests, independent t-tests, chi-square tests, Fisher’s exact tests, and multivariable logistic regression.

**Results:**

The mean knowledge score increased significantly from 57.74 (SD = 11.15) before training to 89.55 (SD = 5.17) afterward (mean difference: 31.81, 95% CI: 28.35–35.27, P < 0.001, Cohen’s d = 3.02). Overall, MDRPI prevalence decreased from 38.5% (95% CI: 24.9–54.1%) pre-intervention to 28.9% (95% CI: 17−44%). However, this difference was not statistically significant using Pearson’s chi-square test (χ^2^ = 0.82, P = 0.365). The distribution of pressure injury stages before implementation was: Stage 1 (28.2%), Stage 2 (7.7%), and Stage 3 (2.6%). Following the intervention, prevalence dropped to Stage 1 (21.1%) and Stage 2 (5.3%), with no new Stage 3 injuries reported. After adjusting for potential confounders (age, Braden score, ICU length of stay, and device exposure duration), implementation of the algorithm was associated with reduced MDRPI occurrence (adjusted OR = 0.42, 95% CI: 0.19–0.91, P = 0.026).

**Conclusion:**

Implementation of the MDRPI prevention algorithm was associated with significant improvement in nurses’ knowledge. A numerically lower prevalence was observed. This suggests that injuries caused by medical equipment are largely absent from current mandatory continuing education or postgraduate nursing curricula, reinforcing the need for a thorough review and the integration of this topic into standard educational programs.

## Introduction

Pressure injuries (PIs) are a serious complication inherent to intensive care unit (ICU) treatment, leading to patient pain and suffering, impaired quality of life, increased healthcare costs, and prolonged hospital stays [1]. In recent years, the National Pressure Injury Advisory Panel (NPIAP) revised the definition of pressure injury to explicitly include Medical Device-Related Pressure Injuries (MDRPI). According to the NPIAP definition, MDRPIs result from the use of devices designed for diagnostic or therapeutic purposes, with the injury pattern typically conforming to the shape or outline of the medical device [2].

The development of pressure injuries poses a significant concern within healthcare institutions due to their profound negative effects on patients and families, including pain, delayed functional recovery, and risk of infection. Furthermore, these injuries lead to prolonged hospitalization, increased costs for institutions, and elevated patient morbidity and mortality [3]. Approximately 20% of ICU patients suffer from MDRPI. The prevalence of MDRPI is particularly concerning in resource-limited settings, underscoring the critical need for focused preventive measures to mitigate the medical burden and improve the quality of life for affected patients [4].

To date, attention dedicated to pressure injuries specifically caused by medical devices remains limited [5]. The likelihood of MDRPI has increased concurrently with technological advancements and the escalating utilization of sophisticated medical equipment on patients [6]. Patients in the ICU are especially susceptible to MDRPI given the necessary and often prolonged use of multiple medical devices [7]. In the ICU setting, PIs may sometimes be overlooked because nurses and specialists often prioritize the pathology and care of other vital organs over skin integrity. However, it is observed that while patients may recover from their primary illnesses, some are left with chronic injuries caused by the hospitalization period, which can persist for months or even years [8]. MDRPI can be considered a key indicator in the assessment of nursing care quality and patient safety [9]. A positive correlation exists between nurses’ knowledge level and the quality of patient care delivered. For instance, a study by Ali et al. identified a highly significant statistical correlation between nurses’ total knowledge scores and their overall performance [10]. Consequently, education is the most crucial step in achieving quality care. Educational interventions are essential for improving nurses’ skills in MDRPI prevention, ensuring a positive impact on patient care outcomes and elevating the overall quality of services provided [11,12]. A recent systematic review by Kim et al. (2023) confirmed the effectiveness of educational interventions for nurses on MDRPI prevention. This study demonstrated that education and active participation of nurses in pressure injury management training courses are vital for enhancing their performance in preventing pressure injuries [13]. The most logical approach to prevent MDRPI is ensuring that nurses possess sufficient knowledge regarding the etiological factors that lead to the development of these injuries [14]. Therefore, determining the current level of nurses’ knowledge concerning medical device-related pressure injuries is an essential foundational step.

Unlike general pressure injuries, there is currently a lack of robust, standardized evidence guiding the prevention and management of MDRPI [4]. Preventive and therapeutic interventions provided to patients with medical device-related PIs are often not systematic, relying instead on routine care practices without clear, evidence-based guidelines [15]. ICU nurses may sometimes fail to recognize MDRPI due to low awareness or knowledge, excessive workload, and varied skill levels. Furthermore, effective pressure injury management can be complicated because nurses often require physician consent or cooperation before adjusting the size or position of a medical device [16]. This highlights the critical need for a structured algorithm that lists logical and sequential activities to ensure both early assessment and effective management of pressure injuries. This study, therefore, investigated the association between implementation of a standardized MDRPI prevention algorithm and nurses’ knowledge and subsequent MDRPI severity in ICU patients.

## Materials and Methods

### Study design and setting

This study was designed as a single-group, pre-test/post-test quasi-experimental study, conducted without randomization or a control group, aimed at evaluating the association between the implementation of an evidence-based medical device-related pressure injury (MDRPI) prevention algorithm and patient outcomes as part of routine clinical practice. The intervention was educational and practice-based in nature and did not involve any experimental therapeutic, pharmacological, or invasive procedures administered to patients. For patient-level analyses, MDRPI prevalence was defined as the proportion of patients with at least one MDRPI. For the analysis of MDRPI location and device types, the unit of analysis was the individual MDRPI lesion, as patients could have multiple lesions.

The study was conducted between February 2025 and April 2025 at the Xxx Educational and Medical Center in Xxx. Data collection took place in the adult General Intensive Care Unit (GICU), which consisted of two units with a total of 28 active beds (16 beds in the first unit and 12 beds in the second).

All methods were performed in accordance with the relevant guidelines and regulations. The study protocol was approved by the Ethics Committee of Shahid Beheshti University of Medical Sciences (Ethics Code: IR.SBMU.PHARMACY.REC.1403.124; approval date: January 15, 2025). Written informed consent was obtained from all participating nurses before enrollment. The ethics committee waived the requirement for written informed consent from patients, as the study involved retrospective chart review for baseline data collection and routine clinical care for the post-intervention period, with no additional experimental procedures. All patient data were anonymized prior to analysis.

### Participants

The study population included eligible patients admitted to the adult GICU and the nurses responsible for providing their care. Sample size was calculated for the primary outcome of nurse knowledge [14] using G*Power software (version 3.1). With an alpha of 0.05, power of 0.80, and effect size of 0.5 for a paired t-test comparing pre- and post-intervention knowledge scores, the required sample was 38 nurses.

For patient outcomes, a convenience sample of 77 patients (39 pre-intervention, 38 post-intervention) was enrolled based on the availability of eligible patients during the study period. No formal a priori sample size calculation was performed for patient outcomes, as these were considered secondary/exploratory analyses. This is acknowledged as a limitation, and the patient outcome findings are interpreted with appropriate caution.

Inclusion criteria for patients were hospitalization in the intensive care unit and the presence of at least one attached medical device. Inclusion criteria for nurses included employment in the intensive care unit and willingness to participate.

Exclusion criteria for patients included conditions that prevented examination of the skin beneath the attached medical device, removal of the medical device, death, or transfer to another department within the first 24 hours. Exclusion criteria for nurses included unwillingness to participate.

### Instruments

Data were collected using demographic forms, the Medical Device-Related Pressure Injury Knowledge Questionnaire (MDRPUKQ) [17], and the MDRPI Prevention Algorithm. The algorithm was adapted from Seong et al. (2021) [16] and included six device types: endotracheal tubes, nasogastric tubes, central venous lines, arterial lines, oxygen masks, and NIV masks. The final algorithm comprises three core steps: identifying the medical device type and its securement method, assessing for the presence of a pressure injury, and delivering device‑specific preventive care. In cases where a pressure injury is detected, the skin at the intended new attachment site must be evaluated beforehand, and a prophylactic dressing should be applied. Subsequently, the algorithm provides device‑specific guidance on repositioning or loosening the device to ensure safe re‑stabilization. Once the device is fixed at the new site, a dressing corresponding to the NPUAP stage is applied to manage the existing injury, and skin status is documented. If no injury is found, the protocol mandates scheduled skin assessments or device repositioning at predetermined intervals, tailored to each device type. To prevent oversight, all devices in use—beyond those specifically listed—are categorized under “other medical devices” to guarantee that skin evaluation is never bypassed. The next phase involves determining the indication for prophylactic dressing, which may be warranted in the presence of impaired sensation, sub‑device moisture, poor perfusion, compromised tissue integrity, malnutrition, or localized/generalized edema. Moreover, given that the device itself constitutes a pressure source, preventive dressings may also be considered based on anatomical location, device purpose, or configuration. Following dressing application and skin condition documentation, the skin is maintained in a clean, dry state, and the algorithm may be iteratively applied as needed [16].

### Validity and reliability of the instruments

The MDRPI-KQ tool was translated, and its face validity was assessed through the opinions of 5 experts from the Faculty of the University of Medical Sciences. In this descriptive survey, content validity ratio (CVR) and content validity index (CVI) were calculated. The overall content validity of the tool was 0.80, and the CVI was 0.99 [17]. The content validity of this research was evaluated by two members of the scientific faculty and a Wound care specialist, and its reliability was assessed using the Cronbach’s alpha coefficient, which was calculated as 0.783.

The construct validity of the MDRPI prevention algorithm was assessed through two rounds of exploratory and confirmatory factor analysis. In the first round, 11 experts participated. In the second round, a test-retest reliability was performed on 5 patients in the wound care department. The construct validity of the algorithm was confirmed using a questionnaire with a 4-point scale. In the second round, 109 patients were enrolled in the study. In terms of practical construct validity, the algorithm was applied to patients’ conditions, and more than three-quarters of them had a history of at least one pressure injury in the last 24 hours. The reliability of the algorithm was also confirmed [16]. The content validity and reliability of this research were assessed by two faculty members and a Wound care specialist.

### Educational intervention

The educational intervention consisted of a comprehensive training program delivered to all participating nurses. The intervention included:

1. **Training Content:** A 90-minute PowerPoint presentation covering the MDRPI prevention algorithm [16], followed by a 30-minute question-and-answer session. Content included:
  - Device-specific prevention strategies for each of the six device types (endotracheal tubes, nasogastric tubes, central venous lines, arterial lines, oxygen masks, and NIV masks)
  - Risk assessment using the Braden and FourScore tools
  - MDRPI staging according to NPIAP guidelines
  - Identification of early signs of MDRPI
  - Documentation and reporting protocols
2. **Training Format:** The training was delivered in-person as an interactive session by a certified wound care nurse with 10 years of ICU experience.
3. **Timing:** Nurses completed the MDRPUKQ questionnaire immediately before and one week after the training. No other educational activities on MDRPI occurred during the study period.

### Intervention fidelity

To ensure consistent implementation of the prevention algorithm, the following fidelity measures were implemented:

Attendance: All 38 eligible nurses attended the full training session (100% attendance).

Compliance Monitoring: A trained research assistant conducted Twice‑weekly audits (total of 12 audits over 6 weeks) of nursing documentation and direct observation of care practices. Each audit assessed adherence to algorithm components using a standardized checklist developed specifically for this study. The checklist included the following core components:

Device identification and risk assessment (4 items)
Skin assessment under devices (4 items)
Device repositioning according to protocol (6 items, specific to each device type)
Prophylactic dressing application when indicated (3 items)
Documentation of skin findings (3 items)

The audit was performed on a random sample of 5 patients per audit (approximately 60 patient-observation events total), with each patient observed for all attached devices (average 3.8 devices per patient, range 2–7 devices).

Adherence Calculation: For each audit, adherence was calculated as the proportion of checklist items correctly performed or documented. The denominator included all applicable checklist items for the devices in use. Missing documentation was treated as non-adherence. The audit results were discussed with nursing staff in monthly feedback sessions to address any gaps.

Adherence Rates: The mean adherence rate across all audits was 87.3% (range: 78–95%). The lowest adherence was observed for prophylactic dressing application (82.1%), while the highest adherence was for skin assessment (91.5%).

### Outcome assessment

**Primary Outcome:** Change in nurses’ knowledge score.

**Secondary Outcomes:** Secondary outcomes included MDRPI prevalence (proportion of patients with at least one MDRPI) and MDRPI severity (stage according to NPIAP guidelines), anatomical location of MDRPI, and device types associated with MDRPI

Nurses’ knowledge was assessed via a self-administered MDRPUKQ. Patient outcomes were assessed by a research assistant who was blinded to the intervention phase. The research assistant was a trained nurse who was not involved in the educational intervention or the routine care of the study patients. Inter-rater reliability for staging was established (kappa = 0.89) before data collection.

### Statistical analysis

Data analysis was performed using SPSS version 26. Descriptive statistics included means and standard deviations for quantitative variables and frequencies and percentages for qualitative variables. Normality of continuous variables was assessed using the Shapiro-Wilk test; all parametric assumptions were met for the primary outcomes.

Inferential analyses included:

- Paired samples t-test for pre-post comparison of nurse knowledge scores
- Independent-samples t-test for between-group comparisons of continuous variables
- Chi-square test for categorical variables (gender distribution)
- Fisher’s exact test for categorical variables with small cell sizes (breathing patterns, MDRPI location, device types)
- For the primary patient outcome of MDRPI prevalence, Pearson’s chi-square test was used to compare proportions between the independent pre- and post-intervention patient cohorts. We used Fisher’s exact test when expected cell frequencies were less than 5.

Multivariable logistic regression was performed to assess the independent association between algorithm implementation and MDRPI occurrence, adjusting for potential confounders selected a priori based on clinical relevance and previous literature (age, Braden score, ICU length of stay, and device exposure duration). Model fit was assessed using the Hosmer-Lemeshow goodness-of-fit test. Multicollinearity was assessed using variance inflation factors (VIF); all VIF values were below 2.0, indicating no significant multicollinearity. Given the sample size (n = 77, with 26 MDRPI events), the model was limited to five covariates to ensure at least 5 events per variable.

## Results

### Nurse characteristics and knowledge assessment

The demographic data for the participating nurses indicated an average professional work experience of mean = 11.39 years (range: 1–25 years). The average experience specifically within the Intensive Care Unit (ICU) was mean = 5.34 years (range: 1–15 years). Regarding education level, 65.8% of the nurses held a Bachelor’s degree, while 34.2% held a Master’s degree.

**Knowledge Scores:** The mean knowledge score regarding MDRPI before the intervention was 57.74 (SD = 11.15), which indicated an average level of knowledge. Following the educational intervention, the mean knowledge score significantly increased to 89.55 (SD = 5.17), indicating a good level of knowledge post-intervention.

A paired-samples t-test demonstrated a statistically significant increase in nurse knowledge following the training, with an average gain of 31.81 units (95% CI: 28.35–35.27, t (37) = 18.42, P < 0.001, Cohen’s d = 3.02) (Table 1).

**Table 1:** Nurse knowledge on the MDRPI prevention algorithm.

Variable	Mean	Standard Deviation	95% CI	Cohen’s d	P-value
Nurse knowledge before algorithm training	57.74	11.15	53.98-61.50	–	<0.001
Nurse knowledge after algorithm training	89.55	5.17	87.85-91.25	3.02	

### Patient characteristics comparison

The comparison between the pre- and post-intervention patient cohorts (N = 77; 39 pre-intervention, 38 post-intervention) for demographic and clinical variables is summarized in Table 2. The cohorts were comparable on all measured characteristics.

**Table 2:** Comparison of patient characteristics before and after intervention.

Characteristic	Pre-intervention (n = 39)	Post-intervention (n = 38)	Test statistic	P-value
Age (years), mean (SD)	62.4 (15.3)	64.1 (14.8)	t(75) = −0.50	0.619
Gender, n (%)			χ^2^ = 1.52	0.217
- Male	14 (35.9%)	19 (50.0%)		
- Female	25 (64.1%)	19 (50.0%)		
ICU length of stay (days), mean (SD)	8.2 (4.1)	7.9 (3.8)	t(75) = 0.33	0.742
Device exposure duration (days), mean (SD)	6.5 (3.2)	6.1 (2.9)	t(75) = 0.57	0.570
Mechanical ventilation days, mean (SD)	4.8 (2.9)	4.5 (2.6)	t(75) = 0.47	0.640
Vasopressor use, n (%)	22 (56.4%)	20 (52.6%)	χ^2^ = 0.11	0.738
Braden Score, mean (SD)	10.55 (1.74)	10.87 (1.61)	t(75) = −0.84	0.401
FourScore, mean (SD)	10.05 (4.12)	9.87 (3.97)	t(75) = 0.19	0.850
Comorbidities, n (%)				
- Diabetes	11 (28.2%)	9 (23.7%)	χ^2^ = 0.20	0.655
- Hypertension	15 (38.5%)	16 (42.1%)	χ^2^ = 0.10	0.748
- Heart disease	9 (23.1%)	8 (21.1%)	χ^2^ = 0.04	0.835
- Respiratory disease	7 (17.9%)	6 (15.8%)	χ^2^ = 0.06	0.803

Gender: Chi-square test confirmed no significant difference between the two groups regarding patient gender (χ^2^ = 1.52, P = 0.217).

Breathing Pattern: Before the intervention, 59.7% of patients were intubated, 36.4% used an oxygen mask, and 3.9% used a non-invasive ventilation (NIV) mask. After the intervention, 63.6% were intubated, 32.5% used an oxygen mask, and 3.9% used an NIV mask. Fisher’s exact test indicated no significant difference in the distribution of breathing patterns between the two measurement points (P = 0.782).

### MDRPI prevalence and location

The overall prevalence of MDRPI decreased from 38.5% (95% CI: 24.9–54.1%) pre-intervention to 28.9% (95% CI: 17–44.8%) post-intervention. Pearson’s chi-square test showed no statistically significant difference between the two independent groups (χ^2^ = 0.82, P = 0.365). The distribution across severity levels is detailed in Tables 3, 4.

**Table 3:** Percentages are calculated from the total number of MDRPI lesions (37 lesions pre-intervention, 26 lesions post-intervention).

Where MDRPI was created	Before intervention (%)	After intervention (%)
Lips	18.9%	26.9%
Nose	24.3%	30.7%
Hand	43.2%	38.4%
Ear	2.7%	0%
Groin	2.7%	3.8%
Leg	5.4%	0%
Chest	2.7%	0%

Fisher’s exact test results showed no significant difference in the location where MDRPI occurred between the two time points (P = 0.413).

**Table 4:** Comparison of the MDRPI-generating device before and after the intervention.

MDRPI generating device	Before intervention (%)	After intervention (%)
CV-LINE	5.4%	0%
Nasogastric tube	21.6%	26.9%
Arteriovenous lines	43.2%	42.3%
Endotracheal tube	24.3%	26.9%
Oxygen mask	2.7%	0%
NIV mask	2.7%	3.8%

Fisher’s exact test results indicated no significant difference between the groups regarding the specific medical device associated with MDRPI formation (P = 0.587).

### Distribution of MDRPI cases (frequency)

- One MDRPI: Remained constant at 26.0% pre- and post-intervention.
- Two MDRPIs: Decreased from 11.7% pre-intervention to 2.6% post-intervention.
- Three MDRPIs: Decreased from 2.6% pre-intervention to 1.3% post-intervention.

### Patient severity scores (Braden and FourScore)

The average patient risk assessment scores showed no significant differences between the pre- and post-intervention measurements.

- FourScore: Pre-intervention: mean = 10.05 (SD = 4.12); Post-intervention: mean = 9.87 (SD = 3.97); P = 0.842
- Braden Score: Pre-intervention: mean = 10.55 (SD = 1.74); Post-intervention: mean = 10.87 (SD = 1.61); P = 0.391

The independent samples t-test confirmed no statistically significant difference in the mean Braden Scores or FourScores between the two time points (Table 5).

**Table 5:** The number of patients with at least one injury of each stage.

Pressure injury stage	Pre-intervention (n = 39)	Post-intervention (n = 38)	P-value
Stage 1	11 (28.2%)	8 (21.1%)	0.599
Stage 2	3 (7.7%)	3 (7.8%)	1.000
Stage 3	1 (2.6%)	0 (0%)	1.000
Stage 4	0%	0%	–

Fisher’s exact test for overall stage distribution: P = 0.625.

### Multivariable analysis

Multivariable logistic regression was performed to assess whether implementation of the prevention algorithm was independently associated with MDRPI occurrence after adjusting for potential confounders. The model included the number of patients: 77 (39 pre-intervention, 38 post-intervention), number of outcome events (MDRPI): 26 (15 pre-intervention, 11 post-intervention), coding of algorithm implementation: 0 = pre-intervention (reference), 1 = post-intervention, and covariates included (selected a priori): age (continuous, years), Braden score (continuous), ICU length of stay (continuous, days), device exposure duration (continuous, days) (Table 6).

**Table 6:** Multivariable logistic regression for factors associated with MDRPI.

Variable	β	SE	Adjusted OR	95% CI	P-value
Algorithm implementation	−0.87	0.39	0.42	0.19-0.92	0.026
Age (per year increase)	0.02	0.02	1.02	0.98-1.06	0.312
Braden Score	−0.16	0.08	0.85	0.72-1.01	0.064
ICU length of stay (per day)	0.08	0.06	1.08	0.96-1.21	0.195
Device exposure duration (per day)	0.11	0.07	1.12	0.98-1.28	0.092

Hosmer-Lemeshow goodness-of-fit: χ^2^ = 5.74, p = 0.676; Nagelkerke R^2^ = 0.181.

After adjusting for potential confounders, implementation of the prevention algorithm remained independently associated with a reduced occurrence of MDRPI (adjusted OR = 0.42, 95% CI: 0.19–0.92, P = 0.026).

## Discussion

This study provides preliminary evidence of an association between implementation of a structured MDRPI prevention algorithm and improved nurse knowledge in the ICU setting. Although a clinically meaningful reduction in MDRPI prevalence was observed (from 38.5% to 28.9%), the difference was not statistically significant in this exploratory analysis.

This finding is consistent with a study conducted in Turkey by Selda Celik et al. [18], which also reported a moderate knowledge level among ICU nurses. In contrast, a study conducted in China indicated that the knowledge level of ICU nurses was higher than in other wards [19]. Following the implementation of the educational algorithm, the nurses’ knowledge level significantly increased, which aligns with the results of a similar study in China that demonstrated the association between education and enhanced nurses’ knowledge and awareness [19] and with a recent systematic review confirming the effectiveness of educational interventions [20]. However, it is important to note that the one-week post-test primarily measures immediate recall rather than long-term knowledge retention.

The most common stage of MDRPI observed in the present study was Stage 1, which is consistent with a Chinese study that also reported Stage 1 as the most frequent stage [7]. However, other studies have reported a higher prevalence of MDRPI in Stage II [18]. Stages 3 and 4 were rare in our study and were treated as emergent cases, receiving daily assessment and treatment. The focus of this study on the severity of the injuries, in addition to prevalence rates, positively influenced nurses’ attitudes and improved their performance. A key aspect of prevention is recognizing progression, as MDRPI progression increases treatment costs and exposes the patient to serious complications [4].

The overall prevalence of MDRPI in the patient cohort decreased from 38.5% pre-intervention to 28.9% post-intervention, which was associated with the implementation of the educational algorithm. This association was further supported by the multivariable analysis; however, these findings should be interpreted with caution given the exploratory nature of this analysis. The relatively small sample size. Although we observed an adjusted OR of 0.42 for the association between algorithm implementation and MDRPI reduction, the wide confidence interval (0.19–0.92) indicates imprecision in the estimate. These findings are hypothesis-generating and require confirmation in larger, adequately powered studies. This post-intervention prevalence is comparable to the 27.2% reported in Turkey [18] but lower than the 58% reported in Egypt [10] and higher than the 13.1% reported in China [7]. A recent meta-analysis by Jackson et al. (2019) [21] reported a pooled MDRPI prevalence of 31% in ICU settings, placing our post-intervention finding of 28.9% within expected ranges. The lower rate observed in our study compared to some regional studies may be attributed to stricter adherence to care protocols post-education.

The most common site for MDRPI in this study was the hand, primarily caused by arterial and venous lines. This finding is attributed to hospital-specific procedures: a low physician-to-patient ratio often necessitates frequent peripheral venous sampling, repeated replacement of central venous lines (CV-LINE), and inherent fragility of peripheral vessels, leading to delayed diagnosis and increased hand injury.

Contrary to our findings, other studies have reported the ear as the most common site [22]. The low rate of ear injuries in our study is likely due to the routine use of preventive measures such as wrapping the oxygen mask elastic and the endotracheal tube with protective foam (vibrille). In a study from China, MDRPI was most prevalent on the finger, followed by the nose [7]. Notably, the routine use of a pulse oximeter and blood pressure cuff while taking vital signs in this study resulted in zero incidence of pressure injury on the finger. Furthermore, nurses were not routinely performing specific preventative measures for lip and nose injuries caused by endotracheal and NG tubes; the new algorithm aimed to integrate appropriate preventive care for these sites moving forward.

A positive correlation was observed between the length of hospital stay and the severity of MDRPI. This is consistent with another study, which indicated that a longer duration of hospitalization leads to the progression of pressure injury stages [23]. Additionally, a positive correlation existed between higher patient age and the prevalence and severity of MDRPI. A study in China identified older age as one of the six risk factors associated with MDRPI [7].

Patients who developed MDRPI had a lower mean FourScore. This observation aligns with a study in Jordan that suggested severe injuries occur at lower levels of consciousness [24]. Similarly, the mean Braden Score was lower in patients with MDRPI. While other studies have associated a low Braden score with MDRPI [25,26], the Braden tool has been considered less reliable for assessing MDRPI risk by some researchers [15,27]. Consistent with the 2019 EPUAP/NPIAP/PPPIA Clinical Practice Guideline [28], we recommend using both Braden and device-specific risk assessment tools. The severity of MDRPI was higher in intubated patients, which is logical given that most oral mucosal and lip MDRPIs stem from the endotracheal tube [15]. In a study of intubated patients in Turkey, 80% experienced MDRPI [29]. Understanding these risk factors is crucial for nurses to effectively manage and prevent MDRPI.

### Comparison with previous studies

The magnitude of improvement in nurse knowledge observed in our study (31.81-point increase, Cohen’s d = 3.02) is larger than that reported in some previous educational interventions. For example, Zhang et al. (2021) [19] reported a 22% improvement in knowledge scores following a similar educational program. This difference may be attributable to the comprehensive nature of our intervention, which included both theoretical and practice-based components, and the use of a structured algorithm rather than general education alone.

Although an observed 9.6-percentage-point reduction in MDRPI prevalence was noted, the difference was not statistically significant in this exploratory analysis and comparable to the 11.5% reduction reported by Seong et al. (2021) [16] in their algorithm implementation study. However, direct comparisons should be made cautiously given differences in study designs and settings.

### Interpretation and cautions

While the findings are encouraging, several factors limit the strength of the conclusions. The single-group pre-post design without a control group means that we cannot establish causality. The observed improvements may be attributable to other factors, including temporal trends in care quality, increased staff awareness (Hawthorne effect), seasonal variation, or concurrent quality improvement activities. Additionally, the short follow-up period (three months) and one-week post-test for knowledge assessment mean that the sustainability of these improvements remains unknown.

## Conclusion

The findings of this study suggest that training based on the MDRPI prevention algorithm was associated with enhanced nurses’ knowledge levels. Interestingly, no correlation was found between the nurses’ knowledge level and their years of education or work experience. This suggests that injuries caused by medical equipment are largely absent from current mandatory continuing education or postgraduate nursing curricula, reinforcing the need for a thorough review and the integration of this topic into standard educational programs.

Implementation of the MDRPI prevention algorithm was associated with a clinically meaningful but statistically nonsignificant reduction in MDRPI prevalence (9.6 percentage points) in this exploratory analysis. These preliminary findings warrant confirmation in larger, adequately powered studies.

Injuries occurring on the face were noted to be the most serious, often progressing rapidly due to the minimal underlying adipose tissue, which can quickly classify them as Stage III pressure injuries. Such severe injuries result in substantial financial burdens for the patient. Consequently, preventing pressure injuries caused by medical devices must be incorporated into mandatory patient safety standards.

Finally, since the MDRPI prevention algorithm currently addresses only six specific types of medical devices, it is recommended that future research focus on developing and expanding this algorithm to create a more comprehensive preventative framework. Future studies should employ randomized controlled designs with longer follow-up periods to establish causality and assess sustainability of improvements.

### Limitations

**Study Design Limitations:** The single-group pre-post design without a control group substantially limits causal inference. We cannot conclude with certainty that the observed improvements in nurse knowledge and patient outcomes were caused by the educational intervention rather than other factors such as:

- Temporal trends in quality of care
- Hawthorne effect (increased awareness due to study participation)
- Seasonal variation in patient characteristics or staffing
- Concurrent quality improvement activities
- Regression to the mean

**Sample and Selection:** The patient outcomes should be considered exploratory. No prospective sample size calculation was conducted for patient outcomes; the available sample was determined by convenience and study period constraints. The absence of a statistically significant difference in MDRPI prevalence between groups should therefore be interpreted in this context.”

**Follow-up and Retention:** The short follow-up period (approximately three months) and one-week post-test for knowledge assessment mean that the sustainability of improvements in knowledge and patient outcomes remains unknown. Long-term knowledge retention and sustained changes in clinical practice were not assessed.

**Intervention Fidelity:** Although we monitored adherence to the algorithm, we cannot confirm that all nurses consistently applied all components of the algorithm throughout the study period. Self-report bias may have influenced compliance documentation.

**Measurement:** The MDRPUKQ questionnaire, while validated, may not capture all aspects of MDRPI knowledge relevant to clinical practice. Staging reliability, while acceptable (kappa = 0.89), may still involve some degree of subjectivity.

### Strengths

A significant strength of this research lies in its focus on the severity of MDRPI progression. Progression of MDRPIs, which can lead to serious complications such as infection and sepsis, further increasing healthcare costs and length of stay, was not a primary focus of previous literature. This study successfully raised nurses’ awareness regarding the critical importance of monitoring and mitigating MDRPI severity to prevent patient deterioration. As such, this appears to be the first study to specifically target the reduction in the severity of MDRPI, rather than solely focusing on prevalence.

Additional strengths include:

- Use of a standardized, evidence-based algorithm adapted from validated sources
- Assessment of both process (nurse knowledge) and outcome (patient) measures
- Blinded outcome assessment by a trained research assistant
- Assessment of intervention fidelity and adherence
- Multivariable analysis to adjust for potential confounders
- Reporting of effect sizes and confidence intervals alongside p-values

## Supporting information

S1 File This file contains the SPSS syntax used for data cleaning, variable recoding, and statistical analyses reported in the study. (DOCX)

S2 File This file contains the Medical Device-Related Pressure Injury Knowledge Questionnaire (MDRPI-KQ) used to assess nurses’ knowledge, including scoring instructions. (DOCX)

S3 Data This file contains the anonymized raw dataset, including demographic information, questionnaire scores, and outcome variables before and after the intervention. (RAR)

## Abbreviations

MDRPI: Medical Device-Related Pressure Injury
ICU: Intensive Care Unit
NPIAP: National Pressure Injury Advisory Panel
EPUAP: European Pressure Ulcer Advisory Panel
PPPIA: Pan Pacific Pressure Injury Alliance
NIV: Non-Invasive Ventilation
SD: Standard Deviation
CI: Confidence Interval
OR: Odds Ratio
