# Supplementary material for: Association of a structured prevention algorithm with nurse knowledge and device-related pressure injury severity in critical care: A pre‑post study
Source: PLoS One. 2026 Oct 27;21(10):e0359842. doi: 10.1371/journal.pone.0359842 (PMC13712620; doi:10.1371/journal.pone.0359842)
Supplement: Supplementary file 1 [file pone.0359842.s001.docx]

**Normality Checks (Shapiro–Wilk)**

spss

* =============================================.

* 1. CHECK NORMALITY FOR CONTINUOUS VARIABLES.

* =============================================.

EXAMINE VARIABLES=Know_Pre Know_Post Age ICU_LOS Device_Exp MV_Days Braden FourScore

/PLOT NONE

/STATISTICS DESCRIPTIVES

/MISSING LISTWISE.

**Nurse Knowledge: Paired t‑test (Primary Outcome)**

spss

* =============================================.

* 2. PAIRED T-TEST FOR NURSE KNOWLEDGE.

* =============================================.

T-TEST PAIRS=Know_Pre WITH Know_Post (PAIRED)

/CRITERIA=CI(.95)

/MISSING=ANALYSIS.

**Patient Baseline Characteristics (Pre vs Post)**

**a) Continuous variables (Age, LOS, Scores, etc.)**

spss

* =============================================.

* 3a. INDEPENDENT T-TESTS FOR CONTINUOUS BASELINE VARIABLES.

* =============================================.

T-TEST GROUPS=Group(0 1)

/MISSING=ANALYSIS

/VARIABLES=Age ICU_LOS Device_Exp MV_Days Braden FourScore

/CRITERIA=CI(.95).

**b) Categorical variables (Gender, Vasopressor, Comorbidities)**

spss

* =============================================.

* 3b. CHI-SQUARE TESTS FOR CATEGORICAL BASELINE VARIABLES.

* =============================================.

CROSSTABS

/TABLES=Group BY Gender Vasopressor Diabetes Hypertension Heart_Disease Resp_Disease

/FORMAT=AVALUE TABLES

/STATISTICS=CHISQ

/CELLS=COUNT ROW

/COUNT ROUND CELL.

**MDRPI Prevalence & Severity (Unadjusted)**

**a) Overall prevalence (patients with ≥1 MDRPI)**

spss

* =============================================.

* 4a. MDRPI PREVALENCE (CHI-SQUARE).

* =============================================.

CROSSTABS

/TABLES=Group BY MDRPI_Any

/FORMAT=AVALUE TABLES

/STATISTICS=CHISQ

/CELLS=COUNT ROW

/COUNT ROUND CELL.

**b) Severity stage distribution (Fisher’s exact due to small cells)**

spss

* =============================================.

* 4b. MDRPI STAGE DISTRIBUTION (FISHER'S EXACT).

* =============================================.

CROSSTABS

/TABLES=Group BY MDRPI_Stage

/FORMAT=AVALUE TABLES

/STATISTICS=FISHER

/CELLS=COUNT ROW

/COUNT ROUND CELL.

**c) Number of MDRPIs per patient (count)**

spss

* =============================================.

* 4c. DISTRIBUTION OF MDRPI COUNT.

* =============================================.

CROSSTABS

/TABLES=Group BY MDRPI_Count

/FORMAT=AVALUE TABLES

/STATISTICS=CHISQ

/CELLS=COUNT ROW

/COUNT ROUND CELL.

**MDRPI Location and Device Type (Lesion-level data)**

spss

* =============================================.

* 5a. ANATOMICAL LOCATION (FISHER'S EXACT).

* =============================================.

CROSSTABS

/TABLES=Group BY Location

/FORMAT=AVALUE TABLES

/STATISTICS=FISHER

/CELLS=COUNT ROW

/COUNT ROUND CELL.

spss

* =============================================.

* 5b. DEVICE TYPE ASSOCIATED WITH MDRPI (FISHER'S EXACT).

* =============================================.

CROSSTABS

/TABLES=Group BY Device

/FORMAT=AVALUE TABLES

/STATISTICS=FISHER

/CELLS=COUNT ROW

/COUNT ROUND CELL.

**Multivariable Logistic Regression (Adjusted OR for MDRPI)**

spss

* =============================================.

* 6. LOGISTIC REGRESSION (ADJUSTED).

* Dependent: MDRPI_Any (0=No, 1=Yes).

* Predictors: Group (0=Pre ref), Age, Braden, ICU_LOS, Device_Exp.

* =============================================.

LOGISTIC REGRESSION VARIABLES MDRPI_Any

/METHOD=ENTER Group Age Braden ICU_LOS Device_Exp

/CONTRAST (Group)=Indicator(1)

/PRINT=CI(95) GOODFIT

/CRITERIA=PIN(0.05) POUT(0.10) ITERATE(20) CUT(0.5).

**Exploratory Correlations (Knowledge Gain vs Experience)**

spss

* =============================================.

* 7. COMPUTE KNOWLEDGE GAIN AND CORRELATE WITH ICU EXPERIENCE.

* =============================================.

COMPUTE Know_Gain = Know_Post - Know_Pre.

EXECUTE.

CORRELATIONS

/VARIABLES=Know_Gain ICU_Exp

/PRINT=TWOTAIL NOSIG

/MISSING=PAIRWISE.

* T-test for knowledge gain by Education level.

T-TEST GROUPS=Education(1 2)

/MISSING=ANALYSIS

/VARIABLES=Know_Gain

/CRITERIA=CI(.95).

spss

* =============================================.

* 8. COMPUTE COHEN'S D FOR PAIRED T-TEST.

* =============================================.

COMPUTE Mean_diff = 31.81. * <-- Replace with your actual mean difference.

COMPUTE SD_diff = 10.75. * <-- Replace with your actual SD of differences.

COMPUTE Cohen_d = Mean_diff / SD_diff.

EXECUTE.

DESCRIPTIVES VARIABLES=Cohen_d

/STATISTICS=MEAN.
