# Supplementary material for: Association of a structured prevention algorithm with nurse knowledge and device-related pressure injury severity in critical care: A pre‑post study
Source: PLoS One. 2026 Oct 27;21(10):e0359842. doi: 10.1371/journal.pone.0359842 (PMC13712620; doi:10.1371/journal.pone.0359842)
Supplement: Supplementary file 2 [file pone.0359842.s002.docx]

Medical Device-Related Pressure Injuries Knowledge Questionnaire (MDRPI-KQ)

| Knowledge items | True | False | No idea |
| --- | --- | --- | --- |
| **Description** |  |  |  |
| 1. MDRPIs are pressure injuries caused by medical devices and equipments. (T) |  |  |  |
| 2. MDRPIs are caused by constant pressure or friction due to medical devices. (T) |  |  |  |
| 3. MDRPIs constitute approximately 1/3 of all pressure injuries that may occur in the hospital environment. (T) |  |  |  |
| 4. In MDRPIs, the tissue around or under the device and equipment takes the shape of the device. (T) |  |  |  |
| 5. The source of pressure in MDRPIs is the medical device itself. (T) |  |  |  |
| 6. MDRPIs usually occur in areas with bony prominences (F). |  |  |  |
| 7. MDRPIs are simple wounds and do not cause serious complications. (F) |  |  |  |
| 8. MDRPI is most common in the sacrum. (F) |  |  |  |
| 9. There are no differences in the appearance of general pressure injuries and MDRPIs. (F) |  |  |  |
| **Risk Factors** | | |  |
| 10. Hypertension is not a risk factor for MDRPIs. (T) |  |  |  |
| 11. MDRPIs are potentially caused only by breathing apparatus and feeding tubes. (F) |  |  |  |
| 12. Hypoalbuminemia and malnutrition are not causes of MDRPIs. (F) |  |  |  |
| 13. All patients with a medical device are at risk for MDRPIs. (T) |  |  |  |
| 14. Patients with signs of localized and generalized edema have a higher risk of MDRPIs. (T) |  |  |  |
| 15. Patients with swallowing problems are at risk of MDRPIs. (T) |  |  |  |
| 16. Face masks used for non-invasive positive pressure ventilation (NIPPV) do not cause MDRPIs. (F) |  |  |  |
| 17. Commonly used risk assessment scales do not assess the risk associated with MDRPIs. (T) |  |  |  |
|  | | |  |
| 18. The most common stage of encountering a MDRPI is stage 2 (T). |  |  |  |
| 19. Considering MDRPIs: if the medical device causes a pressure injury on the mucosa staging is achieved using the “International Pressure Ulcer Classification System” (F). |  |  |  |
| 20. Considering MDRPIs: if the medical device causes a pressure injury on the skin no staging is required (F). |  |  |  |
|  | | |  |
| 21. Placing the medical device or equipment under the immobile patientshould be avoided. (T) |  |  |  |
| 22. MDRPIs do not require any treatment and heal spontaneously. (F) |  |  |  |
| 23. The skin around and under medical devices or equipment should be observed every 48-72 hours for signs of injuries (F). |  |  |  |
| 24. If the patient is at risk of edema or exhibits signs of edema, skin assessment should be performed more frequently than usual. (T) |  |  |  |
| 25. Medical devices except for the endotracheal tube should be relocated periodically to redistribute pressure (F). |  |  |  |
| 26. Medical devices and equipment should be removed from the part of the body they are attached to as soon as medically possible. (T) |  |  |  |
| 27. Relocating the medical devices at regular intervals is the most important preventive method to prevent MDRPIs. (T) |  |  |  |
| 28. The skin surface in the area where the medical device is located should be massaged to prevent MDRPIs. (F) |  |  |  |
| 29. If the dietary plan of adult patients at risk of MDRPIs does not meet nutritional requirements, a protein rich nutritional supplement should be recommended to the patients. (T) |  |  |  |
| 30. Using medical devices in a way that minimizes skin damage (soft material etc.) reduces the risk of injury formation. (T) |  |  |  |
| 31. Medical devices and equipment should be firmly fixed on in order to avoid the risk of dislocation. (F) |  |  |  |
| 32. The medical device should be placed directly on the skin (F) |  |  |  |
| 33. The skin on which medical devices are worn should be regularly moistened with moisturizing products in order to prevent MDRPIs. (F) |  |  |  |
| 34. The skin on which medical devices are worn should be kept clean and dry in order to prevent MDRPIs. (T) |  |  |  |
| 35. Medical devices and equipment should be the appropriate size for the patient. (T) |  |  |  |
| 36. To reduce the pressure associated with the medical device, a dressing/pad should be used under the devices. (T) |  |  |  |

Abbreviations: T: True, F:False

The questionnaire consisted of four sub-dimensions: Description (9 items), Risk Factors (8 items), Staging (3 items) and Prevention and Treatment (16 items). 20 of the total 36 statements included in the questionnaire were affirmative, whereas 16 of them were negative. Each statement in the form aiming to determine the knowledge level of nurses can be answered “true”, “false” or “no idea”. Each “true” answer is scored as 1, whereas “false” and “no idea” answers are scored as 0. The total score that can be obtained from the Information Form varies between 0 and “36” points. An increase in the mean score obtained from the form indicates an increase in the knowledge of nurses about MDRPIs.

The correct answer percentage in the survey is calculated by adding the number of correct answers, dividing this by the total number of questions and then multiplying by 100. A 70% correct response rate (corresponding to 25.2 points) is considered as the threshold for “passing” the test. If the percentage of questions answered correctly is between 80% and 90% (corresponding to a score between 28.8 and 32.4), the result is considered as “good”, while a score higher than 90% (over 32.4 points) is considered as “very good”.
